# *DNAJC3*-Related Syndromic Monogenic Diabetes Without Clinically Evident Neurological Manifestations in an Adult: Expanding the Phenotypic Spectrum

**DOI:** 10.3390/genes17060687

**Published:** 2026-06-11

**Authors:** Norah A. Alshehri, Lemmese Alwatban, Joud S. Almutairi, Dina S. Almunif, Khalid F. Alsadhan, Abdullah A. Alrasheed

**Affiliations:** Department of Family and Community Medicine, College of Medicine, King Saud University (KSU), P.O. Box 2925, Riyadh 11461, Saudi Arabia; lalwatban@ksu.edu.sa (L.A.); jalmutairi1@ksu.edu.sa (J.S.A.); dalmunif@ksu.edu.sa (D.S.A.); kalsadhan@ksu.edu.sa (K.F.A.); aalrasheed1@ksu.edu.sa (A.A.A.)

**Keywords:** *DNAJC3*, monogenic diabetes, consanguinity, sensorineural hearing loss, endoplasmic reticulum stress

## Abstract

**Background/Objectives**: *DNAJC3*-related syndromic monogenic diabetes is a rare autosomal recessive disorder that presents as juvenile-onset non-autoimmune diabetes; it has been associated with sensorineural hearing loss, hypothyroidism, short stature, and variable degrees of neurological manifestations. A delayed diagnosis occurs frequently because of fragmented subspecialty care and lack of awareness of syndromic monogenic diabetes. **Methods**: We report a 34-year-old Saudi male from a consanguineous family with insulin-treated diabetes diagnosed during adolescence. He had long-standing sensorineural hearing loss, hypothyroidism, and short stature, which were managed separately. **Results:** Genetic analysis using whole-exome sequencing identified a homozygous likely pathogenic *DNAJC3* variant, c.1177C>T p.(Arg393*), confirming the diagnosis of *DNAJC3*-related syndromic monogenic diabetes. In addition, he demonstrated no clinically evident neurological manifestations at the time of evaluation, including ataxia, despite reaching adulthood, highlighting the phenotypic variability associated with *DNAJC3*-related disease. **Conclusions:** This case adds to the growing evidence supporting phenotypic variability in *DNAJC3*-related syndromic monogenic diabetes by describing an adult presentation without clinically evident neurological manifestations at the time of evaluation. It highlights how systemic manifestations may remain unrecognized when managed separately across different specialties. In individuals with atypical diabetes accompanied by multisystem involvement, particularly in the setting of consanguinity, early consideration of monogenic diabetes and timely genetic testing may facilitate accurate diagnosis and molecular classification. Establishing a specific genetic diagnosis supports appropriate genetic counseling, informs reproductive decision-making, and may help reduce prolonged diagnostic uncertainty.

## 1. Introduction

Diabetes mellitus (DM) is a heterogeneous metabolic disorder traditionally classified into type 1 diabetes (T1DM), type 2 diabetes (T2DM), gestational diabetes, and other specific types of diabetes [1]. Monogenic diabetes is an increasingly recognized but frequently underdiagnosed form of diabetes; it is caused by defects in a single gene that can either affect pancreatic β-cell function or development, and accounts for up to 3% of all diabetes diagnoses prior to the age of 35 [2].

Due to overlapping clinical features with T1DM and T2DM, 50–90% of monogenic diabetes cases have been reported to be incorrectly diagnosed as one of the two aforementioned conditions, resulting in incorrect treatment and delays in diagnosing the underlying genetic defect [1,2,3].

*DNAJC3* (DnaJ Heat Shock Protein Family Member C3)-related diabetes represents an extremely rare and clinically distinctive form of monogenic diabetes [4,5,6]. The *DNAJC3* gene encodes a co-chaperone of the endoplasmic reticulum (ER) protein BiP, which plays a pivotal role in the regulation of ER stress and the unfolded protein response. Because of their extensive insulin biosynthesis requirements, pancreatic β-cells are highly susceptible to ER stress. Loss of the co-chaperone activity of *DNAJC3* leads to chronic ER stress, subsequent loss of β-cell functionality and ultimately insulin deficiency [7].

The clinical phenotype that has been described with homozygous mutations in the *DNAJC3* gene was initially described by Synofzik et al. (2014) [4]. They provided descriptions of three siblings presenting with juvenile-onset diabetes and multisystem neurodegeneration, which included progressive cerebellar ataxia, upper motor neuron degeneration, polyneuropathy, sensorineural deafness and progressive cerebral atrophy. Exome sequencing revealed that these siblings had a homozygous stop mutation in the *DNAJC3* gene [4]. Subsequent reports expanded the recognized phenotype to include hypothyroidism, short stature, cognitive impairment, pancreatic atrophy, sensorimotor neuropathy, and variable degrees of neurological involvement, demonstrating substantial phenotypic heterogeneity among affected individuals [5,6,7,8]. The condition is inherited in an autosomal recessive pattern (OMIM #616192) and is significantly correlated with consanguinity.

To date, very limited cases have been reported worldwide. Only a small number of these originated from Saudi Arabia [8,9]. Diagnosis is often delayed because the individual manifestations of the syndrome are frequently managed separately by different specialists without recognition of an underlying unifying genetic disorder.

This lack of awareness of the underlying common pathophysiology can lead to delayed diagnosis. Furthermore, the absence of clinically evident neurological manifestations at the time of evaluation in some patients may obscure the clinical picture and reduce the index of suspicion for this condition.

In this paper, we present a 34-year-old male from Saudi Arabia who had long-standing symptoms of multi-system diseases before receiving one unifying genetic diagnosis. Whole-exome sequencing ultimately identified a homozygous *DNAJC3* variant, c.1177C>T p.(Arg393*), confirming the diagnosis of *DNAJC3*-related syndromic monogenic diabetes.

We also describe how this patient’s phenotype contributes additional evidence supporting the expanding clinical spectrum of *DNAJC3*-related disease. Specifically, his adult presentation without clinically evident neurological manifestations at the time of evaluation highlights the phenotypic variability of this disorder and underscores the diagnostic challenges posed by fragmented subspecialty care in individuals with multisystem involvement.

## 2. Case Presentation

### 2.1. Detailed Case Description

The patient is a 34-year-old Saudi man who was born to consanguineous (first cousin) parents. He has been referred to our center in September 2025 for management of longstanding insulin-treated diabetes mellitus with multisystem involvement.

The patient’s early clinical course was managed at external healthcare facilities. Sensorineural hearing loss was diagnosed at 9 years of age and managed with bilateral hearing aids. Recent audiological evaluation at our center confirmed bilateral sensorineural hearing loss. When he was 13 years of age, he developed short stature and after an evaluation for short stature, he started receiving recombinant human growth hormone. While being followed on this regimen, he was found to have diabetes mellitus and was placed on insulin therapy without a definitive classification of diabetes type. There was no history of diabetic ketoacidosis. He also had hypothyroidism diagnosed at the age of 13 years and was treated with levothyroxine. These conditions were each separately managed over many years before a unifying diagnosis was recognized.

At presentation, the patient had persistently poor glycemic control. Upon physical examination, the patient’s height was 147 cm, corresponding to marked short stature (below the 3rd percentile for adult males) and weight was 70 kg, corresponding to a body mass index (BMI) of 32.4 kg/m^2^. No dysmorphic features or acanthosis nigricans were observed. Bilateral hearing aids were in place.

The patient had completed university-level education and demonstrated no clinically apparent cognitive impairment based on history and functional status. No clinically evident neurological manifestations, including ataxia or gait disturbance, were observed at the time of evaluation. Neuroimaging, electrophysiological studies, and standardized neurocognitive assessments were not performed. A summary of the patient’s clinical course and key phenotypic features is presented in Table 1.

His laboratory evaluation included persistent elevations in HbA1c values of 10.5% to 13.7% over previous years. Following optimized treatments, his HbA1c value improved to 8.7% in December 2025 at our center (reference range: <5.7%). Autoimmune markers for diabetes, including anti-glutamic acid decarboxylase (anti-GAD), zinc transporter 8 (ZnT8), and islet cell antibodies, were negative. His serum C-peptide level was 0.39 nmol/L (reference range: 0.37–1.47 nmol/L), indicating detectable endogenous insulin secretion despite longstanding insulin-treated diabetes.

In addition, thyroid function tests demonstrated elevated thyroid-stimulating hormone (TSH) of 7.26 mIU/L (reference range: 0.4–4.0 mIU/L) with low free thyroxine (FT4) of 11.6 pmol/L (reference range: 12–22 pmol/L), consistent with hypothyroidism requiring ongoing levothyroxine therapy.

The renal assessment disclosed an elevated urine albumin-to-creatinine ratio of 1480 mg/g (reference range: <30 mg/g) and an estimated glomerular filtration rate (eGFR) of 76 mL/min/1.73 m^2^ (reference range: >90 mL/min/1.73 m^2^), supporting the presence of diabetic nephropathy. Additionally, ophthalmologic evaluation confirmed advanced diabetic retinopathy.

**Figure 1:**
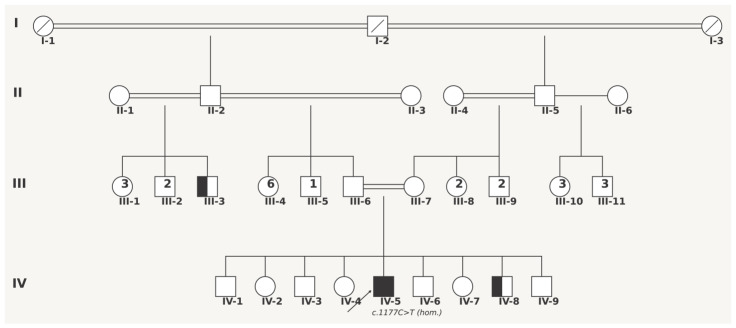
Pedigree of the family. Squares represent males and circles represent females. The arrow indicates the proband (IV-5), who was found to carry the homozygous *DNAJC3* variant c.1177C>T p.(Arg393*). Filled symbols indicate the genetically confirmed proband. Half-filled symbols (left black, right white) indicate individuals with a similar reported clinical phenotype who did not undergo genetic testing. Unfilled symbols indicate unaffected individuals. A diagonal line through a symbol indicates a deceased individual. Double horizontal lines denote consanguineous unions. Numbers within symbols indicate the number of individuals of that sex. Generation I individuals were deceased.

The patient is the fifth of nine siblings born to consanguineous parents. Both parents have diabetes mellitus. One sibling was reported to have a similar clinical phenotype including diabetes mellitus, sensorineural hearing loss, short stature, hypothyroidism, and ataxia. However, no medical records, neurological evaluations, or genetic confirmation were available for review, as the sibling was followed at another institution and his records were not accessible to the investigators. Family genetic testing and segregation analysis were not performed because additional family members declined genetic evaluation despite counseling regarding its potential clinical and familial implications. The family structure and pattern of inheritance are illustrated in Figure 1.

Reassessment of the overall clinical phenotype, including multisystem involvement and consanguinity, together with long-standing insulin-treated diabetes, preserved endogenous insulin secretion, absence of ketoacidosis, and negative autoimmune markers, raised strong suspicion of a syndromic form of monogenic diabetes, prompting genetic investigation.

### 2.2. Molecular Analysis

Whole-exome sequencing (WES) was performed using the CentoXome^®^ MOx 1.0 platform (Centogene GmbH, Rostock, Germany). A peripheral blood sample was collected on 27 July 2025, and the final report was issued on 25 September 2025.

Molecular analysis identified a homozygous likely pathogenic nonsense variant in *DNAJC3*: NM_006260.4:c.1177C>T, p.(Arg393*).

This variant introduces a premature termination codon in exon 10 of 12 and has been previously reported in association with syndromic juvenile-onset diabetes and multisystem involvement [4,5,6,7,8]. Variant interpretation was performed according to ACMG/AMP guidelines [10]. The finding is consistent with autosomal recessive *DNAJC3*-related syndromic diabetes (OMIM #616192). The identified *DNAJC3* variant was considered clinically relevant because it was consistent with the patient’s phenotype and the established autosomal recessive inheritance pattern of *DNAJC3*-related syndromic monogenic diabetes.

A heterozygous likely pathogenic frameshift variant in *CHEK2* (p.His54LeufsTer22) was identified as an incidental finding associated with hereditary cancer predisposition. The patient was referred for genetic counseling and discussion of appropriate surveillance recommendations.

In addition, heterozygous pathogenic variants in *DYSF* and *FKRP* were detected, consistent with carrier status for autosomal recessive neuromuscular disorders. No additional actionable secondary findings were identified according to current ACMG recommendations for the reporting of secondary findings. The mitochondrial genome was included in the whole-exome sequencing platform used in this case, and no clinically relevant mitochondrial variants were identified. A summary of the identified genetic variants and their clinical relevance is provided in Table 2.

## 3. Discussion

This case describes a 34-year-old Saudi male from a consanguineous family in whom whole-exome sequencing identified a homozygous nonsense variant in *DNAJC3* (c.1177C>T, p.(Arg393*)). The patient presented with juvenile-onset diabetes, sensorineural hearing loss, hypothyroidism, and short stature, but without clinically evident neurological manifestations at the time of evaluation. This phenotype adds to the growing body of evidence supporting phenotypic variability in *DNAJC3*-related syndromic monogenic diabetes.

*DNAJC3*-related diabetes is a rare autosomal recessive syndrome characterized by progressive β-cell dysfunction accompanied by variable multisystem involvement, including sensorineural hearing loss, hypothyroidism, growth impairment, and neurological abnormalities [4,7]. Early reports described severe multisystem neurodegeneration with ataxia, neuropathy, cognitive impairment, and abnormal neuroimaging findings [4]. However, subsequent reports have demonstrated substantial variability in the severity and progression of neurological manifestations [8,9].

The first genetically confirmed cases reported by Synofzik et al. demonstrated juvenile-onset diabetes associated with progressive multisystem neurodegeneration, establishing *DNAJC3* deficiency as a syndromic disorder [4]. Subsequent studies, including those by Lytrivi et al., further expanded the phenotype and provided mechanistic evidence linking *DNAJC3* deficiency to endoplasmic reticulum stress-mediated β-cell apoptosis [7].

As summarized in Table 3, previously reported genetically confirmed *DNAJC3* cases consistently exhibited juvenile-onset diabetes, hearing loss, short stature, and endocrine abnormalities, whereas neurological involvement ranged from mild manifestations to severe progressive neurodegeneration. In this context, the present case contributes additional evidence supporting a broader phenotypic spectrum and suggests that clinically apparent neurological manifestations at the time of evaluation may not be apparent in all affected individuals.

A notable feature of this case is the absence of clinically evident neurological manifestations at the time of evaluation despite more than two decades of disease duration. Nevertheless, this observation should be interpreted cautiously. Neuroimaging, electrophysiological studies, and standardized neurocognitive assessments were not performed as part of the clinical evaluation. Consequently, the present findings should be regarded as evidence of phenotypic variability rather than definitive proof of absent neurological involvement.

The prolonged diagnostic delay observed in this patient reflects a common challenge in syndromic monogenic diabetes. Individuals with multisystem manifestations are frequently evaluated by different specialties over many years without recognition of a unifying genetic diagnosis [3]. In the present case, the coexistence of juvenile-onset diabetes, sensorineural hearing loss, hypothyroidism, short stature, negative islet autoantibodies, and preserved C-peptide levels strongly suggested a non-autoimmune etiology and ultimately prompted genetic investigation. Earlier recognition of these characteristic features may facilitate timely diagnosis and reduce prolonged diagnostic uncertainty [2,3]. Whole-exome sequencing was instrumental in establishing the diagnosis in the present case and has become an increasingly valuable tool for identifying rare syndromic forms of monogenic diabetes. However, its limitations should also be recognized. These include the detection of variants of uncertain significance (VUS) and incidental or secondary findings that may require careful interpretation, genetic counseling, and integration with the clinical phenotype to ensure appropriate clinical decision-making.

The patient had developed advanced microvascular complications, including diabetic nephropathy and retinopathy, after more than two decades of diabetes. Although these complications are not specific to *DNAJC3*-related disease, they highlight the substantial long-term disease burden associated with prolonged hyperglycemia. Their presence in the current patient emphasizes the need for careful metabolic follow-up and routine screening for diabetes-related complications in individuals with syndromic forms of diabetes.

**Figure 2:**
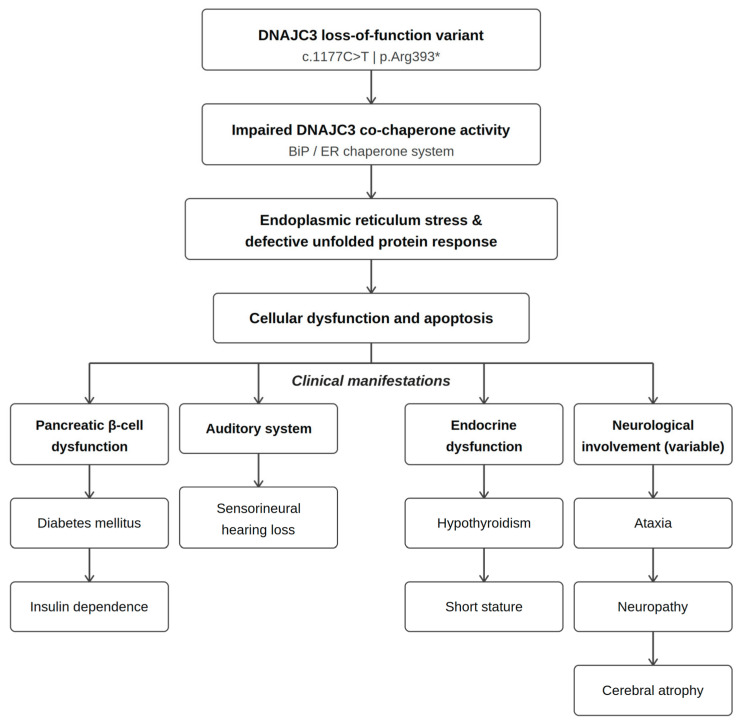
Proposed pathogenic mechanism of *DNAJC3* deficiency and its relationship to β-cell dysfunction and multisystem disease.

*DNAJC3* encodes an endoplasmic reticulum co-chaperone involved in regulation of the unfolded protein response and cellular adaptation to endoplasmic reticulum stress [7]. Loss-of-function variants impair these protective mechanisms, leading to progressive β-cell dysfunction and apoptosis. Experimental studies have demonstrated that *DNAJC3* deficiency promotes β-cell death through activation of endoplasmic reticulum stress pathways and mitochondrial apoptotic signaling, providing a biological explanation for the progressive insulin deficiency observed in affected individuals [11]. The identified nonsense variant is predicted to result in premature truncation of the protein and loss of normal *DNAJC3* function, consistent with the established pathogenic mechanism of the disorder (Figure 2).

These findings further support the role of endoplasmic reticulum stress dysregulation in the pathogenesis of *DNAJC3*-related diabetes and other monogenic disorders involving the unfolded protein response. In addition, they suggest that cellular stress-response pathways may contribute to the phenotypic variability observed among affected individuals [7].

In the present case, diabetes was diagnosed during follow-up after initiation of growth hormone therapy. A similar temporal association has been reported in some previously published *DNAJC3* cases [8]. However, the available evidence does not establish a causal relationship between growth hormone therapy and diabetes onset, and this observation should be interpreted with caution [12]. Further studies are required to determine whether growth hormone therapy contributes to diabetes manifestation in genetically susceptible individuals or whether the observed association reflects the natural progression of the underlying disease.

The mechanisms underlying the marked phenotypic variability observed among reported *DNAJC3* cases remain incompletely understood. Although loss-of-function variants consistently disrupt endoplasmic reticulum homeostasis and β-cell survival, the severity of neurological and endocrine manifestations varies considerably between affected individuals. Potential explanations include genetic modifiers, environmental influences, differences in residual cellular stress responses, and age-dependent penetrance. These factors may contribute to the absence or delayed appearance of clinically evident neurological manifestations at the time of evaluation in some affected individuals despite severe loss-of-function variants.

The differential diagnosis in the present case included several syndromic forms of monogenic diabetes. Mitochondrial diabetes was considered because of the coexistence of diabetes mellitus and sensorineural hearing loss; however, parental consanguinity and the autosomal recessive inheritance pattern favored a nuclear genetic etiology. Wolfram syndrome was also considered, although the absence of optic atrophy and diabetes insipidus made this diagnosis less likely. These considerations highlight the importance of comprehensive genetic testing in patients presenting with diabetes and multisystem involvement.

Several limitations should be acknowledged. First, neuroimaging, electrophysiological studies, and standardized neurocognitive assessments were not performed, and the possibility of ascertainment bias should be considered. Second, segregation analysis was not available because genetic testing of family members was not performed. Although additional relatives exhibited a similar clinical phenotype, confirmation of variant segregation within the family was not possible. Finally, conclusions are inherently limited by the single-patient nature of this report. Nevertheless, this case contributes valuable information regarding the expanding phenotypic spectrum of *DNAJC3*-related syndromic monogenic diabetes and generates hypotheses for future investigations.

## 4. Conclusions

This case contributes additional evidence supporting the expanding phenotypic spectrum of *DNAJC3*-related syndromic monogenic diabetes by describing an adult patient with juvenile-onset diabetes, sensorineural hearing loss, hypothyroidism, and short stature, but without clinically evident neurological manifestations at the time of evaluation. These findings support the recognized phenotypic variability of *DNAJC3*-related disease while acknowledging that neurological involvement cannot be excluded.

The findings highlight the importance of considering monogenic diabetes in individuals with atypical diabetes, multisystem involvement, and a history of consanguinity. In the present case, whole-exome sequencing enabled molecular diagnosis after years of separate management of individual manifestations. Similar approaches may be valuable in selected patients with negative autoimmune markers and preserved C-peptide levels in atypical diabetes and associated multisystem features. Accurate molecular diagnosis supports genetic counseling and informed reproductive decision-making, particularly in populations with a high prevalence of consanguinity.

## Figures and Tables

**Table 1:** Clinical Course and Key Phenotypic Features.

Age	Clinical Feature	Key Findings	Management
9 years	Sensorineural hearing loss	Bilateral SNHL confirmed on audiological assessment	Hearing aids
13 years	Short stature	Endocrine evaluation	Recombinant human growth hormone therapy
13 years	Diabetes mellitus (unclassified)	Hyperglycemia detected during follow-up; no history of DKA	Initiation of insulin therapy
13 years	Hypothyroidism	Elevated TSH with reduced FT4	Levothyroxine replacement
13–33 years	Chronic poor glycemic control	Persistently elevated HbA1c (10.5–13.7%)	Ongoing insulin therapy
34 years	Comprehensive clinical evaluation; Genetic diagnosis	HbA1c reduced to 8.7%; negative islet autoantibodies (anti-GAD, ZnT8, ICA); preserved C-peptide (0.39 nmol/L); markedly elevated UACR (1480 mg/g); reduced eGFR (76 mL/min/1.73 m^2^); advanced retinopathy; Whole-exome sequencing identified homozygous *DNAJC3* variant	Treatment optimization and management of diabetic complications; Confirmation of syndromic monogenic diabetes

Abbreviations: SNHL, sensorineural hearing loss; TSH, thyroid-stimulating hormone; FT4, free thyroxine; DKA, diabetic ketoacidosis; UACR, urine albumin-to-creatinine ratio; eGFR, estimated glomerular filtration rate.

**Table 2:** Summary of Genetic Findings.

Gene	Variant (HGVS)	Zygosity	Classification	Clinical Significance
*DNAJC3*	c.1177C>T p.(Arg393*)	Homozygous	Likely pathogenic	Causative variant for syndromic monogenic diabetes
*CHEK2*	p.(His54LeufsTer22)	Heterozygous	Likely pathogenic	Incidental finding; associated with hereditary cancer predisposition
*DYSF*	c.167dup p.(Ile58HisfsTer8)	Heterozygous	Pathogenic	Carrier status for limb-girdle muscular dystrophy
*FKRP*	c.1364C>A p.(Ala455Asp)	Heterozygous	Pathogenic	Carrier status for muscular dystrophy-related disorder

Abbreviations: HGVS, Human Genome Variation Society.

**Table 3:** Comparative summary of published *DNAJC3* Related Syndromic Monogenic Diabetes Cases and the Present Case.

Genetic Finding (cDNA/Protein)	Age at DM Onset	DM Features	Short Stature	Thyroid Dysfn.	Neurological Features	SNHL	Pancreatic Involvement
**Synofzik et al., 2014 [4] (Am J Hum Genet)**							
c.580C>T p.(Arg194*) Homozygous	18 y	Non-autoimmune; insulin-dependent	+	NR	Ataxia, neuropathy, cognitive deficits, cerebral atrophy	+	NR
c.580C>T p.(Arg194*) Homozygous	18 y	Non-autoimmune; insulin-dependent	+	NR	Ataxia, neuropathy, cognitive deficits	+	NR
c.580C>T p.(Arg194*) Homozygous	15 y	Non-autoimmune; insulin-dependent	+	NR	Ataxia, neuropathy, cognitive deficits	+	NR
del exons 6–12 Homozygous	14 y	Non-autoimmune; insulin-dependent	+	NR	Ataxia, neuropathy, neurodegeneration	+	NR
del exons 6–12 Homozygous	11 y	Non-autoimmune; insulin-dependent	+	NR	Ataxia, neuropathy, neurodegeneration	−	NR
**Bublitz et al., 2017 [5] (J Inherit Metab Dis)**							
c.580C>T p.(Arg194*) Homozygous	19 y	Non-autoimmune; insulin-dependent	+	+	Ataxia, neuropathy, cognitive impairment, MRI abnormalities	+	NR
**Ozon et al., 2020 [6] (Pediatr Diabetes)**							
c.393+2T>G Homozygous	15 y	Hyperinsulinism in infancy → insulin-deficient DM	+	+	Central & peripheral neurodegeneration, ataxia	+	NR
c.393+2T>C Homozygous	13 y	Hyperinsulinism in infancy → insulin-deficient DM	+	+	Central & peripheral neurodegeneration, ataxia	+	NR
**Lytrivi et al., 2021 [7] (Eur J Endocrinol)**							
c.1036C>T p.(Arg346*) + c.1177C>T p.(Arg393*) Compound heterozygous	12 y	Juvenile-onset; insulin-dependent	+	+	Sensorimotor neuropathy, ataxia	+	NR
c.1177C>T p.(Arg393*) Homozygous	16 y	Juvenile-onset; insulin-dependent	+	+	Neurodegeneration	+	NR
**Alwatban et al., 2021 [8] (Front Endocrinol)**							
c.1177C>T p.(Arg393*) Homozygous	11 y	Non-autoimmune; insulin-treated	+	+	Ataxia, neurodegeneration	+	Atrophic pancreas
c.1177C>T p.(Arg393*) Homozygous	14 y	Non-autoimmune; insulin-treated	+	+	Milder ataxia; normal brain MRI at 28 y	+	Atrophic pancreas
**Present Case (2026)**							
c.1177C>T p.(Arg393*) Homozygous	13 y	Non-autoimmune; insulin-treated	+	+	No clinically evident neurological manifestations at the time of evaluation *	+	Not assessed

Abbreviations: DM, diabetes mellitus; SNHL, sensorineural hearing loss; NR, not reported; del, deletion; y, years at onset. Symbols: +, present; −, absent. * At the time of evaluation. Neuroimaging, electrophysiological studies, and formal neurocognitive assessments were not performed; therefore, neurological involvement cannot be excluded.

## Data Availability

The original contributions presented in this study are included in the article. Further inquiries can be directed to the corresponding author.

## Abbreviations

The following abbreviations are used in this manuscript:

**Abbreviation**	**Term**
DM	Diabetes Mellitus
T1DM	Type 1 Diabetes Mellitus
T2DM	Type 2 Diabetes Mellitus
ER	Endoplasmic Reticulum
UPR	Unfolded Protein Response
WES	Whole-Exome Sequencing
GH	Growth Hormone
HbA1c	Hemoglobin A1c
Anti-GAD	Anti–Glutamic Acid Decarboxylase Antibodies
eGFR	Estimated Glomerular Filtration Rate
UACR	Urine Albumin-to-Creatinine Ratio
ACMG	American College of Medical Genetics and Genomics
AMP	Association for Molecular Pathology
OMIM	Online Mendelian Inheritance in Man
MODY	Maturity-Onset Diabetes of the Young

## References

[1] Bonnefond A., Unnikrishnan R., Doria A., Vaxillaire M., Kulkarni R.N., Mohan V., Trischitta V., Froguel P. (2023). Monogenic diabetes. Nat. Rev. Dis. Primers.

[2] Greeley S.A.W., Polak M., Njølstad P.R., Barbetti F., Williams R., Castano L., Raile K., Chi D.V., Habeb A., Hattersley A.T. (2022). ISPAD Clinical Practice Consensus Guidelines 2022: The diagnosis and management of monogenic diabetes in children and adolescents. Pediatr. Diabetes.

[3] Misra S., Owen K.R. (2018). Genetics of monogenic diabetes: Present clinical challenges. Curr. Diabetes Rep..

[4] Synofzik M., Haack T.B., Kopajtich R., Gorza M., Rapaport D., Greiner M., Schönfeld C., Freiberg C., Schorr S., Holl R.W. (2014). Absence of BiP co-chaperone DNAJC3 causes diabetes mellitus and multisystemic neurodegeneration. Am. J. Hum. Genet..

[5] Bublitz S., Alhaddad B., Synofzik M., Kuhl V., Lindner A., Freiberg C., Schmidt H., Strom T., Haack T., Deschauer M. (2017). Expanding the phenotype of DNAJC3 mutations: A case with hypothyroidism additionally to diabetes mellitus and multisystemic neurodegeneration. Clin. Genet..

[6] Ozon Z.A., Alikasifoglu A., Kandemir N., Aydin B., Gonc E.N., Karaosmanoglu B., Celik N.B., Eroglu-Ertugrul N.G., Taskiran E.Z., Haliloglu G. (2020). Novel insights into diabetes mellitus due to DNAJC3 defect: Evolution of neurological and endocrine phenotype in the pediatric age group. Pediatr. Diabetes.

[7] Lytrivi M., Senée V., Salpea P., Fantuzzi F., Philippi A., Abdulkarim B., Sawatani T., Marín-Cañas S., Pachera N., Degavre A. (2021). DNAJC3 deficiency induces β-cell mitochondrial apoptosis and causes syndromic young-onset diabetes. Eur. J. Endocrinol..

[8] Alwatban S., Alfaraidi H., Alosaimi A., Alluhaydan I., Alfadhel M., Polak M., Almutair A. (2021). Case report: Homozygous DNAJC3 mutation causes monogenic diabetes mellitus associated with pancreatic atrophy. Front. Endocrinol..

[9] Alwatban S.M., Alfaraidi H., Alfadhel M., Almutair A.N. (2021). Homozygous DNAJC3 mutation in a Saudi family causing maturity-onset diabetes of the young, hypothyroidism, short stature, neurodegeneration, and hearing loss. Front Genet..

[10] Richards S., Aziz N., Bale S., Bick D., Das S., Gastier-Foster J., Grody W.W., Hegde M., Lyon E., Spector E. (2015). Standards and guidelines for the interpretation of sequence variants: A joint consensus recommendation. Genet. Med..

[11] Fonseca S.G., Gromada J., Urano F. (2011). Endoplasmic reticulum stress and pancreatic β-cell death. Trends Endocrinol. Metab..

[12] Soliman A., Alyafei F., De Sanctis V., Alaaraj N., Hamed N., Ahmed S., Bedair A. (2024). The impact of growth hormone (GH) therapy on glucose metabolism: A narrative review mainly focused on GH-deficient children and adolescents. World J. Adv. Res. Rev..

